# Citation Classics in Cone Beam Computed Tomography: The 100 Top-Cited Articles

**DOI:** 10.1155/2018/9423281

**Published:** 2018-12-30

**Authors:** Shailesh M. Gondivkar, Sachin C. Sarode, Amol R. Gadbail, Rima S. Gondivkar, Nilookumari Choudhary, Shankargouda Patil

**Affiliations:** ^1^Assistant Professor, Department of Oral Medicine and Radiology, Government Dental College & Hospital, Nagpur, Maharashtra, India; ^2^Professor, Department of Oral and Maxillofacial Pathology & Microbiology, Dr. D.Y. Patil Dental College and Hospital, Dr. D.Y. Patil Vidyapeeth, Sant-Tukaram Nagar, Pimpri, Pune, Maharashtra, India; ^3^Assistant Professor, Department of Dentistry, Indira Gandhi Government Medical College, Nagpur, Maharashtra, India; ^4^X Graduate, K. M. Shah Dental College & Hospital, Piparia, Vadodara, Gujarat, India; ^5^Department of Oral and Maxillofacial Pathology & Microbiology, Dr. D.Y. Patil Dental College and Hospital, Dr. D.Y. Patil Vidyapeeth, Sant-Tukaram Nagar, Pimpri, Pune, Maharashtra, India; ^6^Associate Professor, Department of Maxillofacial Surgery and Diagnostic Sciences, Division of Oral Pathology, College of Dentistry, Jazan University, Jazan, Saudi Arabia

## Abstract

**Aim:**

The purpose of this study was to identify and characterize the 100 most cited articles on cone beam computed tomography (CBCT).

**Materials and Methods:**

A comprehensive list of citation classics in CBCT was generated by searching the Scopus database without year or language restrictions. The top 100 articles were retrieved after reading abstracts or full texts. The following study variables were evaluated: number of citations, citation density, journal name, impact factor, category and quartile of journals, publication year, authors with their affiliations, article type, and topics covered.

**Results:**

The citation number ranged from 86 to 624, with a mean of 161.9 citations per article. The top 100 articles were distributed in 29 journals, and the journal with the most articles was Dentomaxillofacial Radiology (*n*=16). The articles were published between 1998 and 2012, and eight authors published more than 3 papers. The USA produced the most papers (*n*=38), followed by the UK (*n*=12). King's college London Dental Institute led the list of classics, with 8 articles.

**Conclusions:**

This is the first citation analysis to provide a detailed list of the most influential articles on CBCT and helps to recognize the quality of the works, discoveries, and trends steering the field.

## 1. Introduction

Dental imaging research has flourished since the inception of dentomaxillofacial radiology as a specialty branch. One of the major breakthroughs is the cone beam computed tomography (CBCT) technique, which involves innovation of tomographic imaging systems and consequent volumetric image reconstruction specifically designed for dental usage. There has been rapidly progressing development in CBCT in recent years due to the emergence of advanced technologies. There have been many articles published on the different aspects and changes that have occurred in the field of CBCT in recent years. Due to the wide range of topics covered on CBCT, it may be valuable for researchers to determine articles with the greatest impact among the published literature. Additionally, the characterization of high-impact studies may help young investigators and clinicians identify research hotspots [1] and could serve as teaching materials for the field.

Citation analysis (CA) is a quantitative analysis of research literature that provides the frequency and pattern of citations [2]. Although its role in quantifying the quality of an article has long been discussed, CA is still the most powerful and commonly used parameter for judging the merit of an influential article or journal in a particular field [3]. CA involves evaluating and ranking an article or journal based on citation count, and this helps identify the milestones completed in understanding the core aspects of a targeted field and emphasizes research outputs, researchers, and future research trends [4]. Therefore, CA can help researchers and organizations to prioritize research areas for delivering support and validate the return on investment in research funding [5].

In recent decades, bibliometric studies have been performed in various subjects and important topics of dentistry such as endodontics, [6] orthodontics, [7] maxillofacial surgery, [8] oral cancer [9], and oral submucous fibrosis [10]. By examining the clinical applications of CBCT, CA can highlight the emergence of advanced techniques and topics. Despite its importance, there is currently no comprehensive study describing the most influential articles in the field of CBCT. Thus, our present analysis was conducted to recognize the 100 most cited articles on CBCT, and we investigated the study characteristics in an effort to acknowledge information and progress in this field.

## 2. Materials and Methods

### 2.1. Data Source

In July 2018, we used the Science Citation Index Expanded (SCI-E) section of Scopus library database (http://www.scopus.com) to identify the top 100 cited articles on CBCT using the medical subject heading term “Cone Beam Computed Tomography” and “CBCT”. There were no limitations on time period, language, type of study, or type of research subjects. The option “Cited by (Highest)” was applied to obtain the comprehensive list of most-to-least cited articles for further analysis. The titles and abstracts of these articles were then appraised to define their relevance to CBCT, and a list of the 100 most cited CBCT articles was then prepared. The mean citation density was calculated by dividing the total number of citations per year since publication of the article. The articles with more number of citation densities were positioned up in the ranking if two or more articles had same number of total citations.

### 2.2. Data Extraction

The data were extracted using the protocol reported in Lim et al. [11], and then the articles were analysed by two authors (SG and AG) to determine the 100 best-cited publications. The following data were retrieved from each study: name of the journal, publication title, publication year, number of citations, citation density, journal impact factor (IF), journal category/categories, quartile (2016 Journal of Citation Reports (JCR): Science Edition), authorship, institution/country of origin of the first author, article type (preclinical, clinical, narrative review, systematic review, and meta-analysis), and topics covered. The articles related to CBCT imaging techniques, image processing, image analysis, and basic science articles were classified as “preclinical”.

In cases of discrepancy between the two authors (SG and AG), a third author (SS) was included in the discussion until consensus was achieved. The current study is based on the data retrieved from the database and their systematic analysis, and no ethics committee approval was obtained. A Pearson product-moment correlation coefficient test was applied to find associations between the IF of a journal and the number of top 100 articles. The statistical analyses were done using SPSS software (version 22.0) for Microsoft Windows.

## 3. Results

The literature search revealed 4000 articles in the Scopus library for the period 1984 to present. There were 3473 published as “articles” and 246 classified as “reviews.” The overall “*h*” index and “*h*” index after removal of self-citations were 90. The 100 top-cited papers were retrieved for further analysis (Table S1).

### 3.1. Citation Analysis

The top 100 cited articles have been cumulatively cited 16,190 times. The median number of citations was 161.9, with a range of 86 (article rank No. 100) to 624 (article rank No. 1). Only eight articles were cited more than 300 times, and a great number (*n*=66) of the articles were cited between 100 and 300 times. The top-cited article was “A new volumetric CT machine for dental imaging based on the cone-beam technique: Preliminary results” published by Mozzo et al. [12] in European Radiology in 1998. The second most cited paper was by Scarfe et al. [13] and received 599 citations. The article explained the clinical applications of CBCT in dental practice and was published in 2006. The third most cited paper was by Ludlow et al. [14] and compared the effective dose for CB Mercuray, New Tom 3G, and i-CAT CBCT machines and suggested the following: (1) CBCT dose varies considerably depending on the device, field of view (FOV), and selected technique factors and (2) full FOV examination i-CAT doses were 3 to 3.3 times greater than New Tom 3G doses, while Mercuray doses were 10.7 to 9.5 times greater.

The review published by Scarfe et al. [13] in Journal of Canadian Dental Association in 2006 had the highest citation density, with 49.91 citations per year. The lowest citation density (5.68) was found for the study entitled “Preoperative application of limited cone beam computerized tomography as an assessment tool before minor oral surgery” published by Nakagawa et al. [15] in International Journal of Oral and Maxillofacial Surgery in 2002. Article rank Nos. 2, 3, 4, 7, and 11 had greater relative impact than did the top-ranked article. Interestingly, all four articles were published after 2006.

### 3.2. Journals Characteristics and Publication Periods

The journal with the highest number of top 100 cited articles was Dentomaxillofacial Radiology, with 16 articles (Table 1). The Journal of Endodontics, American Journal of Orthodontics and Dentofacial Orthopedics and Oral Surgery, Oral Medicine, Oral Pathology, Oral Radiology, and Endodontics each had more than 10 highly cited articles. Except for the International Endodontic Journal (8 articles), the remaining 24 journals contributed fewer than five articles each.

The greatest number of studies among top 100 were published in the subjects of Dentistry, Oral Surgery (*n* = 16), Dentistry (*n* = 13), and Medicine, Radiology, and Nuclear Medicine and Imaging (*n*=13). Of the top 29 journals, 17 (58.62%) were positioned in the first quartile of their category, 8 (27.58%) were in the second, and only 4 (13.79%) were in the third category. The journals Dental Clinics of North America, Journal of Cranio-Maxillofacial Surgery, Journal of Orofacial Orthopedics, and Journal of the California Dental Association were not included in the 2016 JCR: Science Edition.

The IFs for the journals with the top 100 cited publications ranged from 0.514 to 5.133 (mean 2.232). We found 43 of the top 100 articles were published in journals with IFs more than two, and only one article was published in International Journal of Radiation Oncology Biology Physics, which has an IF more than five. This research article by Zhang et al. [16] explained a simple, effective, and computationally efficient algorithm to reduce the metal artefacts in CBCT images. The Journal of the Canadian Dental Association had the lowest IF (0.514) and contributed a single article. We found statistically nonsignificant correlations between the number of top-cited articles and journal IF (*p* > 0.05).

All the articles in the top 100 list were published in English language. The top 100 articles were published in the past two decades, with a peak of publication between 2006 and 2010. In the present analysis, we found that the highest number of articles was published in the year 2009 (*n*=19). The most recent publication was from 2012 (6 articles), and the oldest one was from 1998.

### 3.3. Authors, Institutions, and Countries of Origin

There were more than 250 authors who were associated with the 100 cited articles. The number of authors ranged between 1 (3 papers) and 16 [17]. We found 8 “frequent authors” who holds authorship for three or more of the top-cited articles (Table 2). This list is led by Patel, who published eight of the articles as an author or co-author. This author is also the only person who is the first author of seven of the articles. The most common departmental affiliations of the authors of the top 100 articles was oral radiology department/oral imaging centres (*n*=35). The remaining authors were from other disciplines of dentistry including endodontics, oral and maxillofacial surgery, orthodontics and dentofacial orthopedics, periodontics, prosthodontics, and medical physics.

The country of origin was designated as the country of the first affiliation of the first author. The 100 top-cited articles were originated from 18 countries and the USA (*n*=38) and the UK (*n*=12) were the most prolific (Table 3). All other countries had fewer than 10 highly cited articles.  Table 4 shows the leading institutions in the field of CBCT research. King's College London Dental Institute was found to be the most productive institution, with eight articles, followed by University of North Carolina School of Dentistry and Catholic University of Leuven, both of which contributed six articles. Among the top nine universities or institutions that published top 100 papers on CBCT, there were five located in the USA.

### 3.4. Type of Article and Topics Covered

The article types included publications considered preclinical research (*n*=58), and 17 were clinical studies. The remaining 23 were classified as narrative reviews, and there were only two meta-analyses. The predominant topics covered in the 75 research articles included the following: accuracy of applications of CBCT in various disciplines of dentistry (23%), followed by comparison between CBCT technology and 2D conventional radiographic techniques (14%), comparison between CBCT and conventional CT machines for their accuracy and dose measurements in dentistry (11%) and image analysis, dose and geometric calibration of new CBCT machines (9%).

## 4. Discussion

This is the first bibliometric study summarizing several features of influential articles on CBCT. The literature in the CBCT field is evolving new technology, which justifies the necessity of CA in this field. Understanding the characteristics of highly cited articles on CBCT may be worthwhile for several reasons. First, the findings of the present study could aid young researchers to keep themselves abreast of classic knowledge. Second, trends identified by the current analysis may be of interest to dental clinicians who apply leading imaging techniques in their practice. Third, dental and maxillofacial radiologists can update themselves with knowledge of highly cited articles presented herein. The studies cover important advancements in CBCT, and many may be in their early stages of clinical application. Finally, the findings of the present analysis may help journal editors and reviewers in critically evaluating manuscripts.

It is well known that researchers elect high-impact journals for their manuscript submission and that journals with high IFs attract high-quality papers. This fact is evidenced by the past studies demonstrating positive associations between citation frequency and IF of the journals [18, 19]. However, the findings of our study are in contrast with these reports (*p* > 0.05). There is a recent growing trend of publishing high-end articles in specialty journals over the high IF journals. Other CAs have also reported that highly influential articles are often published in specialty journals [10, 19]. The present analysis noticed similar findings in which 35 of the top-cited articles were published in the specialty journals with relatively low IF. In this context, we feel that publication in a specialty journal leads to increased attention with more views and downloads from the readers in the concerned field.

The articles presented herein were published between 1998 and 2012, which is consistent with developmental history of CBCT. It is widely accepted that time since publication is directly proportional to the citation frequency and that older articles have an advantage. It has also been reported that a minimum 6 to 15 year period is required to attain a sufficient number of citations to become citation classic [6]. However, in the current analysis, the citation rate progressively rose over the last decade, and this represents an increasing consideration in the field of CBCT. We believe that increased numbers of electronic journals with open access options in recent years smoothly enables distribution of the articles in the scientific community. In addition, the availability of multiple social networking sites allows articles to be shared easily, and this increases visibility and positively impacts citation score. It is noteworthy that 9 of the top 10 articles with greatest citation density were published after 2006.

The present bibliometric analysis found the top 100 articles were published in 29 journals. More than one third of the articles were reflected in dental radiology specialty journals, and this result may justify why a few main journals achieved greater attention and dominated the CBCT literature. Therefore, our findings substantiate the application of Bradford's law [20]. This finding of our study is in accordance with other bibliometric analyses [9, 10, 21]. The journals that belong to 100 best-cited articles presented herein do not belong to any specific field of dentistry. Hence, it would not be possible to pin point the exact field of expertise in the present analysis. However, based on the journal's scope, we have broadly categorized the field for the inclusion in the paper.

All the articles included in the present analysis were in English language. The present study characteristics revealed a majority of the highly cited articles originated in the USA. This observation of our study is consistent with the past studies and is in accordance with the reported growing influence of USA in health science research due to the greater number of scientists and the provision of higher financial support to researchers [22–24]. The UK and European countries also showed higher productivity, which supports the phenomenon that “the better the economic ranking of a country, the higher the quantity and quality of its biomedical publications” [25]. Approximately two-thirds of the authors for the highly cited 100 articles were affiliated with nonradiology departments, and this result demonstrates a broad collaboration between oral/dentomaxillofacial radiologists and nonradiologist dental clinicians, medical physicists, and computer scientists. The previous literature in medical imaging also exhibited a similar type of collaboration between researchers/scientists [26]. We believe that young researchers can focus on this concept of broad collaboration of researchers of different relevant fields to produce high impact/quality research.

In terms of article type, the present analysis reported there was a higher number of basic/technical studies than that of clinical research studies. The technical nature of the top-cited articles appears to be unique to imaging and is consistent with the observation of Brinjikji and colleagues [26]. Bibliometric studies in other disciplines of medicine have shown evidence of clinical research dominance [10, 27, 28]. This finding again reflects the dependency of CBCT imaging on advancements in computer science and physics.

Our study has several limitations associated with inherent shortcomings of bibliometric analysis. We retrieved the citation information from Scopus database. Therefore, there is a probability of noninclusion of true “classic” articles that may be available in other databases such as Web of Science and Google Scholar. CA is always associated with the snowball effect, where researchers incline to cite previous highly cited articles [29]. A bibliometric analysis is a method just to quantify recognition of an article in the particular field and does not directly reflect its quality. The time factor can be a major determinant in CA, whereby older articles have an advantage and recent articles may be neglected even if they have high scientific impact. Notwithstanding these noticeable defects, the data generated here do provide insight into the developmental history of CBCT.

## 5. Conclusion

The present analysis is the first reported attempt to recognize 100 top-cited articles in the field of CBCT with in-depth bibliometric analysis. The panorama of the CBCT is rapidly evolving, and bibliometric analyses yield a meaningful aid that can highlight important transitions in this field. The observations of the present study reflect the exciting potential and increasing role of CBCT in clinical practice. These types of analyses may help researchers with limited health care resources and funding agencies to assess the utmost significant research areas in the field to direct the future research trends.

## Figures and Tables

**Table 1:** Top journals with their individual contribution to the 100 most-cited articles on CBCT.

S. no.	Journal name	Impact factor (2016 JCR: Science Edition)	Quartile	Category(ies)	Number of articles in the top 100
1	Dentomaxillofacial Radiology	1.594	2	Dentistry, Radiology, Nuclear Medicine and Imaging	16
2	Journal of Endodontics	2.807	1	Dentistry	13
3	American Journal of Orthodontics and Dentofacial Orthopedics	1.472	2	Dentistry, Oral Surgery, Medicine and Pathology	11
4	Oral Surgery, Oral Medicine, Oral Pathology, Oral Radiology, and Endodontics	1.416	3	Dentistry, Oral Surgery and Medicine	11
5	International Endodontic Journal	3.015	1	Dentistry	8
6	International Journal pf Oral and Maxillofacial Implants	2.263	1	Dentistry, Oral Surgery	4
7	International Journal of Oral and Maxillofacial Surgery	1.918	1	Dentistry, Oral Surgery	4
8	European Journal of Radiology	2.462	2	Medicine, Radiology, Nuclear Medicine and Imaging	4
9	Medical Physics	2.617	1	Medicine, Radiology, Nuclear Medicine and Imaging	4
10	Clinical Oral Implants Research	3.624	1	Dentistry, Oral Surgery	3
11	Clinical Oral Investigations	2.308	2	Dentistry	2
12	Dental Clinics of North America	Na^*∗*^	Na^*∗*^	Na^*∗*^	2
13	Dental Traumatology	1.413	1	Dentistry, Oral Surgery	2
14	American Journal of Neuroradiology	3.55	1	Medicine, Radiology, Nuclear Medicine and Imaging	1
15	British Journal of Radiology	2.05	2	Medicine, Radiology, Nuclear Medicine and Imaging	1
16	Cleft Palate-Craniofacial Journal	1.133	2	Dentistry, Oral Surgery	1
17	European Radiology	3.967	1	Medicine, Radiology, Nuclear Medicine and Imaging	1
18	Implant Dentistry	1.107	2	Dentistry, Oral Surgery	1
19	International Journal of Periodontics and Restorative Dentistry	1.113	1	Dentistry, Oral Surgery and Periodontics	1
20	International Journal of Radiation Oncology Biology Physics	5.133	1	Medicine, Radiology, Nuclear Medicine and Imaging	1
21	Journal of Clinical Periodontology	3.477	1	Dentistry, Periodontics	1
22	Journal of Craniofacial Surgery	0.788	1	Medicine, Otorhinolaryngology, Surgery	1
23	Journal of Cranio-Maxillofacial Surgery	Na^*∗*^	Na^*∗*^	Na^*∗*^	1
24	Journal of Oral and Maxillofacial Surgery	1.916	1	Dentistry, Oral Surgery	1
25	Journal of Orofacial Orthopedics	Na^*∗*^	Na^*∗*^	Na^*∗*^	1
26	Journal of Periodontology	3.03	1	Dentistry, Periodontics	1
27	Journal of the California Dental Association	Na^*∗*^	Na^*∗*^	Na^*∗*^	1
28	Journal of the Canadian dental association	0.514	3	Dentistry	1
29	Orthodontics and Craniofacial Research	1.115	1	Dentistry, Oral Surgery, Orthodontics	1

^*∗*^Not available in the 2016 JCR: Science Edition.

**Table 2:** Authors with greater than 3 articles in the top 100.

Sr. number	Name of author	First author	Co-author	Last author	Total
1	Patel S.	7	1	0	8
2	Jacobs R.	0	5	2	7
3	Scarfe W. C.	3	2	1	6
4	Dawood A.	0	5	0	5
5	Schutyser F.	0	3	2	5
6	Farman A. G.	0	3	1	4
7	Horner K.	1	2	1	4
8	Loubele M.	3	1	0	4

**Table 3:** Country of origin of the 100 top-cited articles on CBCT.

Rank	Country of origin	Number of articles
1	USA	38
2	UK	12
3	Belgium	9
4	Germany	9
5	Brazil	7
6	Japan	7
7	Netherlands	4
8	Finland	2
9	Greece	2
10	Switzerland	2
11	Australia	1
12	Canada	1
13	China	1
14	France	1
15	India	1
16	Italy	1
17	Spain	1
18	Sweden	1

**Table 4:** Institution of origin with 3 or more top-cited articles on CBCT.

Sr. number	Institution/university	Country	Number of articles
1	King's College London Dental Institute	UK	8
2	University of North Carolina School of Dentistry	USA	6
3	Catholic University of Leuven	Belgium	6
4	University of Louisville School of Dentistry	USA	4
5	University of Michigan School of Dentistry	USA	4
6	Case Western Reserve University	USA	3
7	Federal University of Goiás	Brazil	3
8	University of Bern	Switzerland	3
9	University of California	USA	3

## Data Availability

The data of “published papers on CBCT” used to support the findings of this study are included within the supplementary information file.

## Supplementary Materials

Supplementary Materials Table S1: the 100 top-cited articles on CBCT.
