# Supplementary material for: Citation Classics in Cone Beam Computed Tomography: The 100 Top-Cited Articles
Source: Int J Dent. 2018 Dec 30;2018:9423281. doi: 10.1155/2018/9423281 (PMC6332966; doi:10.1155/2018/9423281)
Supplement: Supplementary Materials — Table S1: the 100 top-cited articles on CBCT. [file 9423281.f1.docx]

**Supplementary data:**

**Table S1:** The 100 top-cited articles on CBCT

| **Rank** | **Article** | **Scopus citations** | **Scopus citation density** |
| --- | --- | --- | --- |
|  | Mozzo, P., Procacci, C., Tacconi, A., Tinazzi Martini, P., Bergamo Andreis, I.A. A new volumetric CT machine for dental imaging based on the cone-beam technique: Preliminary results (1998) European Radiology, 8 (9), pp. 1558-1564. | 624 | 31.2 |
|  | Scarfe, W.C., Farman, A.G., Sukovic, P. Clinical applications of cone-beam computed tomography in dental practice (2006) Journal of the Canadian Dental Association, 72 (1), pp. 75-80. | 599 | 49.91 |
|  | Ludlow, J.B., Davies-Ludlow, L.E., Brooks, S.L., Howerton, W.B. Dosimetry of 3 CBCT devices for oral and maxillofacial radiology: CB Mercuray, NewTom 3G and i-CAT (2006) Dentomaxillofacial Radiology, 35 (4), pp. 219-226. | 476 | 39.66 |
|  | Ludlow, J.B., Ivanovic, M. Comparative dosimetry of dental CBCT devices and 64-slice CT for oral and maxillofacial radiology  (2008) Oral Surgery, Oral Medicine, Oral Pathology, Oral Radiology and Endodontology, 106 (1), pp. 930-938. | 472 | 47.2 |
|  | Arai, Y., Tammisalo, E., Iwai, K., Hashimoto, K., Shinoda, K. Development of a compact computed tomographic apparatus for dental use (1999) Dentomaxillofacial Radiology, 28 (4), pp. 245-248. | 398 | 20.94 |
|  | De Vos, W., Casselman, J., Swennen, G.R.J. Cone-beam computerized tomography (CBCT) imaging of the oral and maxillofacial region: A systematic review of the literature (2009) International Journal of Oral and Maxillofacial Surgery, 38 (6), pp. 609-625. | 364 | 19.15 |
|  | Scarfe, W.C., Farman, A.G. What is Cone-Beam CT and How Does it Work? (2008) Dental Clinics of North America, 52 (4), pp. 707-730. | 354 | 35.4 |
|  | Lascala, C.A., Panella, J., Marques, M.M. Analysis of the accuracy of linear measurements obtained by cone beam computed tomography (CBCT-NewTom) (2004) Dentomaxillofacial Radiology, 33 (5), pp. 291-294. | 303 | 21.64 |
|  | Cotton, T.P., Geisler, T.M., Holden, D.T., Schwartz, S.A., Schindler, W.G. Endodontic Applications of Cone-Beam Volumetric Tomography (2007) Journal of Endodontics, 33 (9), pp. 1121-1132. | 296 | 26.90 |
|  | Sarment, D.P., Sukovic, P., Clinthorne, N. Accuracy of implant placement with a stereolithographic surgical guide (2003) International Journal of Oral and Maxillofacial Implants, 18 (4), pp. 571-577. | 271 | 18.06 |
|  | Pauwels, R., Beinsberger, J., Collaert, B., Theodorakou, C., Rogers, J., Walker, A., Cockmartin, L., Bosmans, H., Jacobs, R., Bogaerts, R., Horner, K. Effective dose range for dental cone beam computed tomography scanners (2012) European Journal of Radiology, 81 (2), pp. 267-271. | 263 | 43.83 |
|  | Patel, S., Dawood, A., Pitt Ford, T., Whaites, E. The potential applications of cone beam computed tomography in the management of endodontic problems (2007) International Endodontic Journal, 40 (10), pp. 818-830. | 261 | 23.72 |
|  | Loubele, M., Bogaerts, R., Van Dijck, E., Pauwels, R., Vanheusden, S., Suetens, P., Marchal, G., Sanderink, G., Jacobs, R.  Comparison between effective radiation dose of CBCT and MSCT scanners for dentomaxillofacial applications (2009) European Journal of Radiology, 71 (3), pp. 461-468. | 252 | 28.00 |
|  | Lofthag-Hansen, S., Huumonen, S., Gröndahl, K., Gröndahl, H.-G. Limited cone-beam CT and intraoral radiography for the diagnosis of periapical pathology (2007) Oral Surgery, Oral Medicine, Oral Pathology, Oral Radiology, and Endodontics, 103 (1), pp. 114-119. | 237 | 21.54 |
|  | Estrela, C., Bueno, M.R., Leles, C.R., Azevedo, B., Azevedo, J.R. Accuracy of Cone Beam Computed Tomography and Panoramic and Periapical Radiography for Detection of Apical Periodontitis (2008) Journal of Endodontics, 34 (3), pp. 273-279. | 230 | 23.00 |
|  | Kobayashi, K., Shimoda, S., Nakagawa, Y., Yamamoto, A. Accuracy in measurement of distance using limited cone-beam computerized tomography (2004) International Journal of Oral and Maxillofacial Implants, 19 (2), pp. 228-231. | 225 | 16.07 |
|  | Schulze, D., Heiland, M., Thurmann, H., Adam, G. Research: Radiation exposure during midfacial imaging using 4- and 16-slice computed tomography, cone beam computed tomography systems and conventional radiography (2004) Dentomaxillofacial Radiology, 33 (2), pp. 83-86. | 224 | 16.00 |
|  | Sukovic, P. Cone beam computed tomography in craniofacial imaging (2003) Orthodontics and Craniofacial Research, 6 (SUPPL1), pp. 31-36. | 222 | 15.85 |
|  | Roberts, J.A., Drage, N.A., Davies, J., Thomas, D.W. Effective dose from cone beam CT examinations in dentistry (2009) British Journal of Radiology, 82 (973), pp. 35-40. | 208 | 23.11 |
|  | Guerrero, M.E., Jacobs, R., Loubele, M., Schutyser, F., Suetens, P., van Steenberghe, D. State-of-the-art on cone beam CT imaging for preoperative planning of implant placement (2006) Clinical Oral Investigations, 10 (1), pp. 1-7. | 199 | 16.58 |
|  | Patel, S. New dimensions in endodontic imaging: Part 2. Cone beam computed tomography (2009) International Endodontic Journal, 42 (6), pp. 463-475. | 196 | 21.77 |
|  | Hashimoto, K., Arai, Y., Iwai, K., Araki, M., Kawashima, S., Terakado, M. A comparison of a new limited cone beam computed tomography machine for dental use with a multidetector row helical CT machine (2003) Oral Surgery, Oral Medicine, Oral Pathology, Oral Radiology, and Endodontics, 95 (3), pp. 371-377. | 194 | 12.93 |
|  | Misch, K.A., Yi, E.S., Sarment, D.P. Accuracy of cone beam computed tomography for periodontal defect measurements (2006) Journal of Periodontology, 77 (7), pp. 1261-1266. | 191 | 15.91 |
|  | Tsiklakis, K., Donta, C., Gavala, S., Karayianni, K., Kamenopoulou, V., Hourdakis, C.J. Dose reduction in maxillofacial imaging using low dose Cone Beam CT (2005) European Journal of Radiology, 56 (3), pp. 413-417. | 190 | 14.61 |
|  | Van Assche, N., Van Steenberghe, D., Guerrero, M.E., Hirsch, E., Schutyser, F., Quirynen, M., Jacobs, R. Accuracy of implant placement based on pre-surgical planning of three-dimensional cone-beam images: A pilot study (2007) Journal of Clinical Periodontology, 34 (9), pp. 816-821. | 179 | 16.27 |
|  | Cevidanes, L.H.S., Styner, M.A., Proffit, W.R. Image analysis and superimposition of 3-dimensional cone-beam computed tomography models (2006) American Journal of Orthodontics and Dentofacial Orthopedics, 129 (5), pp. 611-618. | 179 | 14.91 |
|  | Meyer, E., Raupach, R., Lell, M., Schmidt, B., Kachelrieß, M. Normalized metal artifact reduction (NMAR) in computed tomography(2010) Medical Physics, 37 (10), pp. 5482-5493. | 178 | 22.25 |
|  | Hilgers, M.L., Scarfe, W.C., Scheetz, J.P., Farman, A.G. Accuracy of linear temporomandibular joint measurements with cone beam computed tomography and digital cephalometric radiography (2005) American Journal of Orthodontics and Dentofacial Orthopedics, 128 (6), pp. 803-811. | 175 | 13.46 |
|  | Silva, M.A.G., Wolf, U., Heinicke, F., Bumann, A., Visser, H., Hirsch, E. Cone-beam computed tomography for routine orthodontic treatment planning: A radiation dose evaluation (2008) American Journal of Orthodontics and Dentofacial Orthopedics, 133 (5), pp. 640.e1-640.e5. | 174 | 17.4 |
|  | Patel, S., Dawood, A., Whaites, E., Pitt Ford, T. New dimensions in endodontic imaging: Part 1. Conventional and alternative radiographic systems (2009) International Endodontic Journal, 42 (6), pp. 447-462. | 173 | 19.22 |
|  | Aboudara, C., Nielsen, I., Huang, J.C., Maki, K., Miller, A.J., Hatcher, D. Comparison of airway space with conventional lateral headfilms and 3-dimensional reconstruction from cone-beam computed tomography (2009) American Journal of Orthodontics and Dentofacial Orthopedics, 135 (4), pp. 468-479. | 166 | 18.44 |
|  | Cevidanes, L.H.S., Bailey, L.J., Tucker Jr., G.R., Styner, M.A., Mol, A., Phillips, C.L., Proffit, W.R., Turvey, T. Superimposition of 3D cone-beam CT models of orthognathic surgery patients (2005) Dentomaxillofacial Radiology, 34 (6), pp. 369-375. | 166 | 12.76 |
|  | Suomalainen, A., Kiljunen, T., Käser, Y., Peltola, J., Kortesniemi, M. Dosimetry and image quality of four dental cone beam computed tomography scanners compared with multislice computed tomography scanners (2009) Dentomaxillofacial Radiology, 38 (6), pp. 367-378. | 163 | 18.11 |
|  | Nair, M.K., Nair, U.P. Digital and Advanced Imaging in Endodontics: A Review (2007) Journal of Endodontics, 33 (1), pp. 1-6. | 157 | 14.27 |
|  | Walker, L., Enciso, R., Mah, J. Three-dimensional localization of maxillary canines with cone-beam computed tomography (2005) American Journal of Orthodontics and Dentofacial Orthopedics, 128 (4), pp. 418-423. | 156 | 12.00 |
|  | Matherne, R.P., Angelopoulos, C., Kulild, J.C., Tira, D. Use of Cone-Beam Computed Tomography to Identify Root Canal Systems In Vitro (2008) Journal of Endodontics, 34 (1), pp. 87-89. | 152 | 15.2 |
|  | Swennen, G.R.J., Schutyser, F. Three-dimensional cephalometry: Spiral multi-slice vs cone-beam computed tomography (2006) American Journal of Orthodontics and Dentofacial Orthopedics, 130 (3), pp. 410-416. | 152 | 12.66 |
|  | Miracle, A.C., Mukherji, S.K. Conebeam CT of the head and neck, part 2: Clinical applications (2009) American Journal of Neuroradiology, 30 (7), pp. 1285-1292. | 148 | 16.44 |
|  | Tantanapornkul, W., Okouchi, K., Fujiwara, Y., Yamashiro, M., Maruoka, Y., Ohbayashi, N., Kurabayashi, T. A comparative study of cone-beam computed tomography and conventional panoramic radiography in assessing the topographic relationship between the mandibular canal and impacted third molars (2007) Oral Surgery, Oral Medicine, Oral Pathology, Oral Radiology and Endodontology, 103 (2), pp. 253-259. | 148 | 13.45 |
|  | Marmulla, R., Wörtche, R., Mühling, J., Hassfeld, S. Geometric accuracy of the NewTom 9000 cone beam CT (2005) Dentomaxillofacial Radiology, 34 (1), pp. 28-31. | 147 | 11.30 |
|  | Low, K.M.T., Dula, K., Bürgin, W., von Arx, T. Comparison of Periapical Radiography and Limited Cone-Beam Tomography in Posterior Maxillary Teeth Referred for Apical Surgery (2008) Journal of Endodontics, 34 (5), pp. 557-562. | 146 | 14.6 |
|  | Stratemann, S.A., Huang, J.C., Maki, K., Miller, A.J., Hatcher, D.C. Comparison of cone beam computed tomography imaging with physical measures (2008) Dentomaxillofacial Radiology, 37 (2), pp. 80-93. | 142 | 14.2 |
|  | Tyndall, D.A., Price, J.B., Tetradis, S., Ganz, S.D., Hildebolt, C., Scarfe, W.C. Position statement of the American Academy of Oral and Maxillofacial Radiology on selection criteria for the use of radiology in dental implantology with emphasis on cone beam computed tomography (2012) Oral Surgery, Oral Medicine, Oral Pathology and Oral Radiology, 113 (6), pp. 817-826. | 139 | 23.16 |
|  | Mah, P., Reeves, T.E., McDavid, W.D. Deriving Hounsfield units using grey levels in cone beam computed tomography (2010) Dentomaxillofacial Radiology, 39 (6), pp. 323-335. | 135 | 16.85 |
|  | Tyndall, D.A., Rathore, S. Cone-Beam CT Diagnostic Applications: Caries, Periodontal Bone Assessment, and Endodontic  Applications (2008) Dental Clinics of North America, 52 (4), pp. 825-841. | 132 | 13.2 |
|  | Yang, K., Kwan, A.L.C., Miller, D.F., Boone, J.M. A geometric calibration method for cone beam CT systems (2006) Medical Physics, 33 (6), pp. 1695-1706. | 130 | 10.83 |
|  | Paula-Silva, F.W.G.d., Wu, M.-K., Leonardo, M.R., Bezerra da Silva, L.A., Wesselink, P.R. Accuracy of Periapical Radiography and Cone-Beam Computed Tomography Scans in Diagnosing Apical Periodontitis Using Histopathological Findings as a Gold Standard(2009) Journal of Endodontics, 35 (7), pp. 1009-1012. | 125 | 13.88 |
|  | Suomalainen, A., Vehmas, T., Kortesniemi, M., Robinson, S., Peltola, J. Accuracy of linear measurements using dental cone beam and conventional multislice computed tomography (2008) Dentomaxillofacial Radiology, 37 (1), pp. 10-17. | 123 | 12.3 |
|  | Zhang, Y., Zhang, L., Zhu, X.R., Lee, A.K., Chambers, M., Dong, L. Reducing metal artifacts in cone-beam CT images by preprocessing projection data (2007) International Journal of Radiation Oncology Biology Physics, 67 (3), pp. 924-932. | 123 | 11.18 |
|  | Hatcher, D.C., Dial, C., Mayorga, C. Cone beam CT for pre-surgical assessment of implant sites. (2003) Journal of the California Dental Association, 31 (11), pp. 825-833. | 123 | 8.2 |
|  | Mischkowski, R.A., Pulsfort, R., Ritter, L., Neugebauer, J., Brochhagen, H.G., Keeve, E., Zöller, J.E. Geometric accuracy of a newly developed cone-beam device for maxillofacial imaging (2007) Oral Surgery, Oral Medicine, Oral Pathology, Oral Radiology and Endodontology, 104 (4), pp. 551-559. | 121 | 11 |
|  | Schulze, R.K.W., Berndt, D., D'Hoedt, B.On cone-beam computed tomography artifacts induced by titanium implants(2010) Clinical Oral Implants Research, 21 (1), pp. 100-107. | 119 | 14.87 |
|  | Aranyarachkul, P., Caruso, J., Gantes, B., Schulz, E., Riggs, M., Dus, I., Yamada, J.M., Crigger, M. Bone density assessments of dental implant sites: 2. Quantitative cone-beam computerized tomography (2005) International Journal of Oral and Maxillofacial Implants, 20 (3), pp. 416-424. | 119 | 9.15 |
|  | Garrett, B.J., Caruso, J.M., Rungcharassaeng, K., Farrage, J.R., Kim, J.S., Taylor, G.D. Skeletal effects to the maxilla after rapid maxillary expansion assessed with cone-beam computed tomography (2008) American Journal of Orthodontics and Dentofacial Orthopedics, 134 (1), pp. 8.e1-8.e11. | 118 | 11.8 |
|  | Estrela, C., Bueno, M.R., Azevedo, B.C., Azevedo, J.R., Pécora, J.D. A New Periapical Index Based on Cone Beam Computed Tomography(2008) Journal of Endodontics, 34 (11), pp. 1325-1331. | 117 | 11.7 |
|  | Araki, K., Maki, K., Seki, K., Sakamaki, K., Harata, Y., Sakaino, R., Okano, T., Seo, K. Characteristics of a newly developed dentomaxillofacial X-ray cone beam CT scanner (CB MercuRay™): System configuration and physical properties (2004) Dentomaxillofacial Radiology, 33 (1), pp. 51-59. | 117 | 8.35 |
|  | Benavides, E., Rios, H.F., Ganz, S.D., An, C.-H., Resnik, R., Reardon, G.T., Feldman, S.J., Mah, J.K., Hatcher, D., Kim, M.-J., Sohn, D.-S., Palti, A., Perel, M.L., Judy, K.W.M., Misch, C.E., Wang, H.-L. Use of cone beam computed tomography in implant dentistry: The international congress of oral implantologists consensus report (2012) Implant Dentistry, 21 (2), pp. 78-86. | 115 | 19.16 |
|  | Siewerdsen, J.H., Jaffray, D.A. Cone-beam computed tomography with a flat-panel imager: Effects of image lag (1999) Medical Physics, 26 (12), pp. 2635-2647. | 115 | 6.05 |
|  | Baumgaertel, S., Palomo, J.M., Palomo, L., Hans, M.G. Reliability and accuracy of cone-beam computed tomography dental measurements (2009) American Journal of Orthodontics and Dentofacial Orthopedics, 136 (1), pp. 19-25. | 114 | 12.66 |
|  | Patel, S., Dawood, A., Wilson, R., Horner, K., Mannocci, F. The detection and management of root resorption lesions using intraoral radiography and cone beam computed tomography - an in vivo investigation (2009) International Endodontic Journal, 42 (9), pp. 831-838. | 112 | 12.44 |
|  | Horner, K., Islam, M., Flygare, L., Tsiklakis, K., Whaites, E. Basic principles for use of dental cone beam computed tomography: Consensus guidelines of the European Academy of Dental and Maxillofacial Radiology (2009) Dentomaxillofacial Radiology, 38 (4), pp. 187-195. | 110 | 12.22 |
|  | Swennen, G.R.J., Mollemans, W., De Clercq, C., Abeloos, J., Lamoral, P., Lippens, F., Neyt, N., Casselman, J., Schutyser, F. A cone-beam computed tomography triple scan procedure to obtain a three-dimensional augmented virtual skull model appropriate for orthognathic surgery planning (2009) Journal of Craniofacial Surgery, 20 (2), pp. 297-307. | 110 | 12.22 |
|  | Patel, S., Dawood, A., Mannocci, F., Wilson, R., Pitt Ford, T. Detection of periapical bone defects in human jaws using cone beam computed tomography and intraoral radiography (2009) International Endodontic Journal, 42 (6), pp. 507-515. | 107 | 11.88 |
|  | Loubele, M., Guerrero, M.E., Jacobs, R., Suetens, P., Van Steenberghe, D. A comparison of jaw dimensional and quality assessments of bone characteristics with cone-beam CT, spiral tomography, and multi-slice spiral CT (2007) International Journal of Oral and Maxillofacial Implants, 22 (3), pp. 446-454. | 107 | 9.72 |
|  | Loubele, M., Maes, F., Schutyser, F., Marchal, G., Jacobs, R., Suetens, P. Assessment of bone segmentation quality of cone-beam CT versus multislice spiral CT: a pilot study (2006) Oral Surgery, Oral Medicine, Oral Pathology, Oral Radiology and Endodontology, 102 (2), pp. 225-234. | 106 | 8.83 |
|  | Holberg, C., Steinhäuser, S., Geis, P., Rudzki-Janson, I.Cone-beam computed tomography in orthodontics: Benefits and limitations (2005) Journal of Orofacial Orthopedics, 66 (6), pp. 434-444. | 106 | 8.15 |
|  | Stavropoulos, A., Wenzel, A. Accuracy of cone beam dental CT, intraoral digital and conventional film radiography for the detection of periapical lesions. An ex vivo study in pig jaws (2007) Clinical Oral Investigations, 11 (1), pp. 101-106. | 104 | 9.45 |
|  | Aboul-Hosn Centenero, S., Hernández-Alfaro, F. 3D planning in orthognathic surgery: CAD/CAM surgical splints and prediction of the soft and hard tissues results - Our experience in 16 cases (2012) Journal of Cranio-Maxillofacial Surgery, 40 (2), pp. 162-168. | 103 | 17.16 |
|  | Neelakantan, P., Subbarao, C., Ahuja, R., Subbarao, C.V., Gutmann, J.L. Cone-beam computed tomography study of root and canal morphology of maxillary first and second molars in an Indian population (2010) Journal of Endodontics, 36 (10), pp. 1622-1627. | 103 | 12.87 |
|  | Bernardes, R.A., de Moraes, I.G., Húngaro Duarte, M.A., Azevedo, B.C., de Azevedo, J.R., Bramante, C.M. Use of cone-beam volumetric tomography in the diagnosis of root fractures (2009) Oral Surgery, Oral Medicine, Oral Pathology, Oral Radiology and Endodontology, 108 (2), pp. 270-277. | 102 | 11.33 |
|  | Baratto Filho, F., Zaitter, S., Haragushiku, G.A., de Campos, E.A., Abuabara, A., Correr, G.M. Analysis of the Internal Anatomy of Maxillary First Molars by Using Different Methods (2009) Journal of Endodontics, 35 (3), pp. 337-342. | 102 | 11.33 |
|  | Carter, L., Farman, A.G., Geist, J., Scarfe, W.C., Angelopoulos, C., Nair, M.K., Hildebolt, C.F., Tyndall, D., Shrout, M. American Academy of Oral and Maxillofacial Radiology executive opinion statement on performing and interpreting diagnostic cone beam computed tomography (2008) Oral Surgery, Oral Medicine, Oral Pathology, Oral Radiology and Endodontology, 106 (4), pp. 561-562. | 102 | 10.2 |
|  | Harris, D., Horner, K., Gröndahl, K., Jacobs, R., Helmrot, E., Benic, G.I., Bornstein, M.M., Dawood, A., Quirynen, M. E.A.O. guidelines for the use of diagnostic imaging in implant dentistry 2011. A consensus workshop organized by the European Association for Osseointegration at the Medical University of Warsaw (2012) Clinical Oral Implants Research, 23 (11), pp. 1243-1253. | 100 | 16.66 |
|  | Hirsch, E., Wolf, U., Heinicke, F., Silva, M.A.G. Dosimetry of the cone beam computed tomography Veraviewepocs 3D compared with the 3D Accuitomo in different fields of view (2008) Dentomaxillofacial Radiology, 37 (5), pp. 268-273. | 100 | 10 |
|  | Meyer, E., Raupach, R., Lell, M., Schmidt, B., Kachelrieß, M. Frequency split metal artifact reduction (FSMAR) in computed tomography (2012) Medical Physics, 39 (4), pp. 1904-1916. | 99 | 16.5 |
|  | Hamada, Y., Kondoh, T., Noguchi, K., Iino, M., Isono, H., Ishii, H., Mishima, A., Kobayashi, K., Seto, K. Application of limited cone beam computed tomography to clinical assessment of alveolar bone grafting: A preliminary report (2005) Cleft Palate-craniofacial Journal, 42 (2), pp. 128-137. | 99 | 7.61 |
|  | Scarfe, W.C. Use of cone-beam computed tomography in endodontics joint position statement of the American Association of Endodontists and the American Academy of Oral and Maxillofacial Radiology (2011) Oral Surgery, Oral Medicine, Oral Pathology, Oral Radiology and Endodontology, 111 (2), pp. 234-237. | 97 | 13.85 |
| 1. R | Angelopoulos, C., Thomas, S., Hechler, S., Parissis, N., Hlavacek, M. Comparison Between Digital Panoramic Radiography and Cone-Beam Computed Tomography for the Identification of the Mandibular Canal as Part of Presurgical Dental Implant Assessment(2008) Journal of Oral and Maxillofacial Surgery, 66 (10), pp. 2130-2135. | 97 | 9.7 |
|  | Cohenca, N., Simon, J.H., Roges, R., Morag, Y., Malfaz, J.M. Clinical indications for digital imaging in dento-alveolar trauma. Part 1: Traumatic injuries (2007) Dental Traumatology, 23 (2), pp. 95-104. | 96 | 8.72 |
|  | Hassan, B., Metska, M.E., Ozok, A.R., van der Stelt, P., Wesselink, P.R. Comparison of Five Cone Beam Computed Tomography Systems for the Detection of Vertical Root Fractures (2010) Journal of Endodontics, 36 (1), pp. 126-129. | 95 | 11.87 |
|  | Fortin, T., Champleboux, G., Bianchi, S., Buatois, H., Coudert, J.-L. Precision of transfer of preoperative planning for oral implants based on cone-beam CT-scan images through a robotic drilling machine: An in vitro study (2002) Clinical Oral Implants Research, 13 (6), pp. 651-656. | 95 | 5.93 |
|  | Plooij, J.M., Maal, T.J.J., Haers, P., Borstlap, W.A., Kuijpers-Jagtman, A.M., Bergé, S.J. Digital three-dimensional image fusion processes for planning and evaluating orthodontics and orthognathic surgery. A systematic review (2011) International Journal of Oral and Maxillofacial Surgery, 40 (4), pp. 341-352. | 93 | 13.28 |
|  | Blattner, T.C., George, N., Lee, C.C., Kumar, V., Yelton, C.D.J. Efficacy of Cone-Beam Computed Tomography as a Modality to Accurately Identify the Presence of Second Mesiobuccal Canals in Maxillary First and Second Molars: A Pilot Study (2010) Journal of Endodontics, 36 (5), pp. 867-870. | 93 | 11.62 |
|  | Ballrick, J.W., Palomo, J.M., Ruch, E., Amberman, B.D., Hans, M.G. Image distortion and spatial resolution of a commercially available cone-beam computed tomography machine (2008) American Journal of Orthodontics and Dentofacial Orthopedics, 134 (4), pp. 573-582. | 93 | 9.3 |
|  | Baba, R., Ueda, K., Okabe, M. Using a flat-panel detector in high resolution cone beam CT for dental imaging (2004) Dentomaxillofacial Radiology, 33 (5), pp. 285-290. | 92 | 6.57 |
|  | Nakagawa, Y., Kobayashi, K., Ishii, H., Mishima, A., Ishii, H., Asada, K., Ishibashi, K. Preoperative application of limited cone beam computerized tomography as an assessment tool before minor oral surgery (2002) International Journal of Oral and Maxillofacial Surgery, 31 (3), pp. 322-327. | 91 | 5.68 |
|  | Patel, S., Ricucci, D., Durak, C., Tay, F. Internal root resorption: A review (2010) Journal of Endodontics, 36 (7), pp. 1107-1121. | 90 | 11.25 |
|  | Haney, E., Gansky, S.A., Lee, J.S., Johnson, E., Maki, K., Miller, A.J., Huang, J.C. Comparative analysis of traditional radiographs and cone-beam computed tomography volumetric images in the diagnosis and treatment planning of maxillary impacted canines(2010) American Journal of Orthodontics and Dentofacial Orthopedics, 137 (5), pp. 590-597. | 90 | 11.25 |
|  | Ghaeminia, H., Meijer, G.J., Soehardi, A., Borstlap, W.A., Mulder, J., Bergé, S.J. Position of the impacted third molar in relation to the mandibular canal. Diagnostic accuracy of cone beam computed tomography compared with panoramic radiography (2009) International Journal of Oral and Maxillofacial Surgery, 38 (9), pp. 964-971. | 90 | 10.00 |
|  | Patel, S., Kanagasingam, S., Pitt Ford, T. External Cervical Resorption: A Review (2009) Journal of Endodontics, 35 (5), pp. 616-625. | 90 | 10.00 |
|  | Braut, V., Bornstein, M.M., Belser, U., Buser, D. Thickness of the anterior maxillary facial bone wall-a retrospective radiographic study using cone beam computed tomography (2011) International Journal of Periodontics and Restorative Dentistry, 31 (2), pp. 125-131. | 89 | 12.71 |
|  | Boeddinghaus, R., Whyte, A. Current concepts in maxillofacial imaging (2008) European Journal of Radiology, 66 (3), pp. 396-418. | 89 | 8.9 |
|  | Draenert, F.G., Coppenrath, E., Herzog, P., Müller, S., Mueller-Lisse, U.G. Beam hardening artefacts occur in dental implant scans with the NewTom® cone beam CT but not with the dental 4-row multidetector CT (2007) Dentomaxillofacial Radiology, 36 (4), pp. 198-203. | 89 | 8.09 |
|  | Cohenca, N., Simon, J.H., Mathur, A., Malfaz, J.M. Clinical indications for digital imaging in dento-alveolar trauma. Part 2: Root resorption (2007) Dental Traumatology, 23 (2), pp. 105-113. | 89 | 8.09 |
|  | Honda, K., Larheim, T.A., Maruhashi, K., Matsumoto, K., Iwai, K. Osseous abnormalities of the mandibular condyle: Diagnostic reliability of cone beam computed tomography compared with helical computed tomography based on an autopsy material (2006) Dentomaxillofacial Radiology, 35 (3), pp. 152-157. | 89 | 7.41 |
|  | Sherrard, J.F., Rossouw, P.E., Benson, B.W., Carrillo, R., Buschang, P.H. Accuracy and reliability of tooth and root lengths measured on cone-beam computed tomographs (2010) American Journal of Orthodontics and Dentofacial Orthopedics, 137 (4 SUPPL.), pp. S100-S108. | 88 | 11 |
|  | Palomo, J.M., Rao, P.S., Hans, M.G. Influence of CBCT exposure conditions on radiation dose (2008) Oral Surgery, Oral Medicine, Oral Pathology, Oral Radiology and Endodontology, 105 (6), pp. 773-782. | 88 | 8.8 |
|  | Durack, C., Patel, S., Davies, J., Wilson, R., Mannocci, F. Diagnostic accuracy of small volume cone beam computed tomography and intraoral periapical radiography for the detection of simulated external inflammatory root resorption (2011) International Endodontic Journal, 44 (2), pp. 136-147. | 87 | 12.42 |
|  | Zhang, R., Yang, H., Yu, X., Wang, H., Hu, T., Dummer, P.M.H. Use of CBCT to identify the morphology of maxillary permanent molar teeth in a Chinese subpopulation (2011) International Endodontic Journal, 44 (2), pp. 162-169. | 87 | 12.42 |
|  | Wu, M.-K., Shemesh, H., Wesselink, P.R. Limitations of previously published systematic reviews evaluating the outcome of endodontic treatment (2009) International Endodontic Journal, 42 (8), pp. 656-666. | 86 | 9.55 |
